# London Prices of Drugs.—Feb. 26

**Published:** 1814-03

**Authors:** 


### LONDON PRICES OF DRUGS.—Feb. 26.

*(To be continued regularly.)*

| | £. | s. | d. | £. | s. | d. per |
|---|---|---|---|---|---|---|
| Aloes Barbadoes, from | 20 | 10 | 0 to | 21 | 0 | 0 C. |
| ——Cape | 10 | 0 | 0.. | 11 | 0 | 0 — |
| ——Succotrina | 20 | 0 | 0.. | 0 | 0 | 0 — |
| ——Epatica or E.I. | 10 | 0 | 0.. | 12 | 0 | 0 — |
| Angelica Root | 10 | 0 | 0.. | 12 | 0 | 0 — |
| Alkanet Root | 8 | 0 | 0.. | 0 | 0 | 0 — |
| Antimony Crude | 3 | 15 | 0.. | 4 | 0 | 0 — |
| Aqua Fortis | S. | 0 | 8..D. | 1 | 1 | lb |
| Arrow Root fine | 0 | 2 | 0.. | 0 | 3 | 6 — |
| —— ordinary | 0 | 1 | 0.. | 0 | 1 | 6 — |
| Arsenic Red | 5 | 15 | 0.. | 0 | 0 | 0 C. |
| —— White | 3 | 5 | 0.. | 0 | 0 | 0 — |
| Balsam Capivi | 0 | 5 | 6.. | 0 | 0 | 0 lb. |
| —— Peru | 0 | 19 | 0.. | 1 | 0 | 0 — |
| —— Tolu | 0 | 12 | 0.. | 0 | 13 | 0 — |
| Bark Jesuits Com. | 0 | 3 | 6.. | 0 | 4 | 0 — |
| —— Second | 0 | 6 | 0.. | 0 | 7 | 0 — |
| —— Quil or best | 0 | 8 | 0.. | 0 | 10 | 0 — |
| —— Red | 0 | 7 | 0.. | 0 | 10 | 0 — |
| —— Yellow | 0 | 4 | 6.. | 0 | 5 | 0 — |
| Borax refined E.I. | none. | | | | | |
| —— English | 0 | 3 | 6.. | 0 | 3 | 8 lb. |
| ——unrefined or Tinc. | 10 | 0 | 0.. | 11 | 0 | 0 C. |
| Camphire refined | 0 | 8 | 9.. | 0 | 9 | 0 lb. |
| —— unrefined | 28 | 0 | 0.. | 32 | 0 | 0 C. |
| Cantharides | 0 | 11 | 0.. | 0 | 11 | 3 — |
| Cardamoms (best) | 0 | 7 | 6.. | 0 | 0 | 0 lb. |
| Cassia Buds | 12 | 0 | 0.. } | 0 | 0 | 0 C. |
| —— Fistula W.I. | 12 | 0 | 0.. } | in bond | | — |
| —— Lignea | 12 | 0 | 0.. } | 0 | 0 | 0 — |
| Castorum American | 2 | 10 | 0.. | 2 | 15 | 0 lb. |
| —— Russia | 11 | 0 | 0.. | 0 | 0 | 0 — |
| Castor Oil per bottle 1½ lb. | 0 | 3 | 9.. | 0 | 4 | 3 bo |
| Cocculus Indicus | 3 | 0 | 0.. | 0 | 0 | 0 C. |
| Colocynth Turkey | 0 | 5 | 0.. | 0 | 5 | 6 lb. |
| Columbo Root | 2 | 15 | 0.. | 3 | 0 | 0 C. |
| Cream of Tartar | 1 | 10 | 0.. | 9 | 0 | 0 — |
| Gallangal East-India | 5 | 0 | 0.. | 0 | 0 | 0 C. |
| Gentian Root | 3 | 15 | 0.. | 4 | 0 | 0 — |
| Ginseng | 0 | 1 | 6.. | 0 | 0 | 0 lb. |
| Grains of Guinea | 5 | 10 | 0.. | 6 | 0 | 0 C. |
| Gum Ammo. Drop | 30 | 0 | 0.. | 0 | 0 | 0 — |
| —— Lump | 8 | 0 | 0.. | 9 | 0 | 0 — |
| —— Animi | 3 | 10 | 0.. | 7 | 10 | 0 — |
| —— Arabic E.I. | 2 | 0 | 0.. | 4 | 0 | 0 — |
| —— Turkey Fine | 8 | 10 | 0.. | 9 | 9 | 0 — |
| —— Barbary | 6 | 0 | 0.. | 7 | 0 | 0 — |
| —— Assafœtida | 10 | 0 | 0.. | 15 | 0 | 0 — |
| —— Benjamin | 8 | 10 | 0.. | 35 | 0 | 0 — |
| —— Cambogium | 20 | 0 | 0.. | 26 | 0 | 0 — |
| —— Copal scraped | 0 | 2 | 0.. | 0 | 3 | 6 lb. |
| —— Galbanum | 16 | 0 | 0.. | 20 | 0 | 0 C. |

| | £. | s. | d. | £. | s. | d. per |
|---|---|---|---|---|---|---|
| Gum Guaiacum, from | 0 | 2 | 6 to | 0 | 3 | 9 lb. |
| —— Mastic | 0 | 4 | 6.. | 0 | 4 | 9 — |
| —— Myrrh | 15 | 0 | 0.. | 18 | 0 | 0 C. |
| —— Olibanum | 6 | 0 | 0.. | 7 | 0 | 0 — |
| —— Oppoponax | 80 | 0 | 0.. | 0 | 0 | 0 — |
| —— Sandrac | 6 | 10 | 0.. | 8 | 0 | 0 — |
| —— Seneca Garbled | 5 | 2 | 0.. | 5 | 5 | 0 — |
| —— Tragacanth | 36 | 0 | 0.. | 38 | 0 | 0 — |
| Jalap | 0 | 5 | 0.. | 0 | 5 | 3 lb. |
| Ipecacuanha | 0 | 19 | 0.. | 1 | 0 | 0 — |
| Isinglass Book | 0 | 10 | 6.. | 0 | 11 | 0 — |
| —— Leaf | 0 | 10 | 6.. | 0 | 11 | 0 — |
| —— Long Staple | 0 | 10 | 6.. | 0 | 11 | 0 — |
| —— Short Staple | 0 | 10 | 6.. | 0 | 11 | 0 — |
| Manna Flakey | 0 | 6 | 0.. | 0 | 6 | 6 — |
| —— Sicily in Sorts | 0 | 4 | 0.. | 0 | 5 | 6 — |
| Musk China | 0 | 18 | 0.. | 1 | 0 | 0 oz. |
| Nux Vomica | 3 | 0 | 0.. | 3 | 10 | 0 C. |
| Oil of Vitriol | 0 | 0 | 3¾ | 0 | 0 | 0 lb. |
| Opium East-India | 0 | 19 | 6.. | 1 | 0 | 0 — |
| —— Turkey | 1 | 9 | 0.. | 1 | 10 | 0 — |
| Pink Root | 0 | 4 | 0.. | 0 | 4 | 6 — |
| Quicksilver | 0 | 4 | 10.. | 4 | 11 | 0 — |
| Rhubarb East-India | 0 | 3 | 0.. | 0 | 5 | 0 — |
| —— Russia | 0 | 18 | 0.. | 1 | 0 | 0 — |
| Saffron Spanish | 2 | 5 | 0.. | 2 | 10 | 0 — |
| —— French | 1 | 15 | 0.. | 2 | 0 | 0 — |
| Sago | 6 | 0 | 0.. | 6 | 10 | 0 C. |
| Sal. Ammoniac | 10 | 10 | 0.. | 11 | 0 | 0 — |
| Sarsaparilla | 0 | 3 | 0.. | 0 | 3 | 6 lb. |
| Sassafras | 8 | 0 | 0.. | 9 | 0 | 0 T. |
| Scammony Aleppo | 1 | 6 | 0.. | 1 | 7 | 0 lb. |
| —— Smyrna | 0 | 10 | 0.. | 0 | 12 | 0 — |
| Senna | 0 | 3 | 0.. | 0 | 0 | 0 — |
| Seeds Anni Alicant | 6 | 0 | 0.. | 6 | 10 | 0 C. |
| —— Coriander English | 1 | 10 | 0.. | 1 | 16 | 0 — |
| —— Cummin | 7 | 0 | 0.. | 7 | 10 | 0 — |
| —— Fenugreek | 1 | 10 | 0.. | 1 | 13 | 0 — |
| Shellack | 7 | 0 | 0.. | 8 | 8 | 0 — |
| Sticklack | 6 | 0 | 0.. | 8 | 0 | 0 — |
| Snake Root | 0 | 0 | 0.. | 0 | 0 | 0 lb. |
| Soap Castile or Spanish | 12 | 0 | 0.. | 14 | 0 | 0 C. |
| Spermacceti refined | 0 | 2 | 7.. | 0 | 0 | 0 lb. |
| Tamarinds West-India | 8 | 10 | 0.. | 9 | 0 | 0 C. |
| Tapioca Lisbon | 0 | 0 | 8.. | 0 | 1 | 0 lb. |
| Turmeric Bengal | 3 | 3 | 0.. | 0 | 0 | 0 C. |
| —— China | 4 | 5 | 0.. | 4 | 15 | 0 — |
| —— West-India | 3 | 0 | 0.. | 9 | 0 | 0 — |
| Verdigris Wet | 0 | 3 | 6.. | 0 | 0 | 0 lb. |
| —— Dry | 0 | 5 | 6.. | 0 | 5 | 8 — |
| —— Crystallized | 0 | 8 | 6.. | 0 | 8 | 9 — |
| Vitriol Roman | 0 | 0 | 7.. | 0 | 0 | 0 — |
| —— Foreign white | 4 | 0 | 0.. | 4 | 10 | 0 C. |

Hirudines 15s. per Doz. at Covent Garden Market.

*Price of Vials per Gross.*—8 oz. 70s.—6 oz. 58s.—4 oz. 47s.—3 oz. 43s.—2 oz. 36s.—1 oz. 30s.—½ oz. 24s.

### OBSERVATIONS.

At recent Sales of Merchandize by Auction, Mr. E. Rodwell sold, on the 18th of February, Cassia Fistula, 77s. to 78s. 6d. per Cwt.—2 Casks of Cream of Tartar, 8l. per Cwt.—3 Casks of Antimony, 67s. 6d. to 75s. per Cwt.—5 Barrels of Arrow Root, 11d. to 12d. per lb.—and 1 Bale of Senna, 3s. 10s. per lb.

